# Impact of the Haptic Virtual Reality Simulator on Dental Students’ Psychomotor Skills in Preclinical Operative Dentistry

**DOI:** 10.3390/clinpract12010003

**Published:** 2021-12-28

**Authors:** Abeer Farag, Danya Hashem

**Affiliations:** 1 Department of Restorative Dental Science, College of Dentistry, Taibah University, Madinah P.O. Box 42353, Saudi Arabia; abeerfarag2002@yahoo.com; 2 Department of Restorative Dentistry, Faculty of Dentistry, Minia University, Minia P.O. Box 61519, Egypt

**Keywords:** haptics, virtual reality, simulation, psychomotor skills, pre-clinical operative dentistry, cavity preparation

## Abstract

One of the current trends in dental education is to empower dental students on a global platform using advanced technology. Haptic virtual reality simulation (HVRS) is a relatively new technology in the field of teaching and learning operative dentistry. This study aims to assess the impact of haptic virtual reality simulation (HVRS) on dental students’ psychomotor skills acquisition in preclinical operative dentistry. Class I cavity preparations (CP) were performed at baseline by 21 novice dental students on plastic teeth. Duration of CP was recorded and cavity features were evaluated and scored. Then, students were exposed to HVRS training on CP. Another Class I CP was performed by each student on plastic teeth after HVRS training, then evaluated, and the duration was recorded. There was a statistically significant decrease in CP performance time after HVRS training (*p* < 0.001) and an increase in the mean total marks of CP after HVRS training (*p* < 0.001). The change in the students’ performance in the CP displayed a statistically significant improvement after HVRS training in smoothness of the pulpal floor (*p* = 0.047), pulpal floor direction (*p* = 0.029), buccal, lingual, and mesial wall direction (*p* = 0.004, *p* = 0.025, *p* = 0.002), mesial and distal wall smoothness (*p* = 0.01, *p* = 0.001), internal line angle (*p* = 0.024), and internal point angle (*p* = 0.029). Overall improved performance in psychomotor skills was found after HVRS training. It could be beneficial to incorporate HVRS training early in pre-clinical operative dentistry courses as an adjunct to conventional phantom head training.

## 1. Introduction

Dental students’ acquisition of psychomotor skills is the core feature and main competency of preclinical operative dentistry and the area where the majority of preclinical teaching time is devoted. Globally, dental curricula allocate more time for practicing and enhancing psychomotor skills rather than didactic or theoretical teaching [1].

The most widespread approach in teaching psychomotor skills is the traditional approach lecture/demonstration method. In these traditional methods, the teaching content is delivered as a package of information; the teacher tells the students and shows them how to do the task. The students practice the task in the lab then evaluate their work with the teacher according to a definite detailed criterion-referenced rubric. So clinically unacceptable errors may be encountered more frequently after they are made especially during the initial stage of psychomotor skills acquisition. The student relies on instructor feedback and availability, and may not readily develop skills of self-assessment and critical thinking [2].

Currently, dental schools use the phantom head simulator for teaching psychomotor skills in preclinical operative dentistry using plastic teeth which is considered the mainstay simulation since its existence. Unfortunately, plastic teeth do not simulate enamel and dentine hardness. No pathosis (caries) exist in plastic teeth and it is difficult for the teacher to explain the real tactile sensation [3,4].

There have been recent developments in virtual-reality technology, haptics, and robotics in dental simulation, which provide more optimal practice conditions to smoothen the transition from the traditional model-based simulation laboratory to the clinic. These technologies help create an environment in which users can practice clinical procedures in the different disciplines of dentistry [5]. For example in Japan, a robot patient was developed, which can provide real-life simulations such as coughing, shaking neck, tongue thrusting, and salivary secretion which was found to enhance dental skills and improve management of dental emergencies in a dental setting [6,7]. Furthermore, virtual reality simulation offers the prospect of creating a digital environment for its users to perform various exercises such as cavity preparation (by providing multiple magnified images), caries excavation, and light-curing techniques [8]. 

Most recently, combining haptic technology with virtual reality simulation introduced haptic virtual reality simulators; a cutting-edge technology that revolutionized dental education globally [8]. Haptic virtual reality simulators provide a sense (haptic) of feedback through the device being held by the user in the form of sounds, pressure, and vibrations. The goal is to simulate an optimal and genuine sense of the clinical procedure, providing a learning experience that resembles reality [9].

This kind of haptic simulation in pre-clinical operative dentistry provides sensory feedback of preparation in enamel and dentine, as well as caries excavation [3]. In addition, it provides standardized cases, objective assessment, and interactivity. Furthermore, it stimulates the use of reflective forms of assessment that involve students in a self-assessment process to identify individual learning needs and self-directed learning [10]. It was claimed that adjunctive training in these simulators seems to enhance students’ learning and psychomotor skill acquisition, provide unlimited access to practice clinical tasks, and reduce the required faculty supervision time [11,12].

The haptic virtual reality simulation (HVRS) is a relatively new technology in the field of teaching and learning operative dentistry with new data emerging in the literature. However, no studies have been conducted in Saudi Arabia on the use of HVRS on dental students in pre-clinical operative dentistry. Therefore, this study aims to assess the impact of HVRS on Saudi dental students’ psychomotor skills acquisition in preclinical operative dentistry by comparing the quality of cavities prepared by novice students on plastic teeth for the first time before exposure to HVRS training with the quality of cavities prepared on plastic teeth after a period of practicing on the HVRS. 

## 2. Materials and Methods

This prospective single-arm interventional study was carried out at Taibah University, College of Dentistry female section, Madinah, Saudi Arabia and was reviewed and approved by Taibah University, College of Dentistry Research Ethics Committee with reference number (TUCDREC/20190505). The sample size was determined where the minimal sample size to detect a change in the cavity preparation score by 10% (minimum meaningful change) with a power of 80% was calculated to be 15 students. All third-year female dental students (*n* = 21) with a mean age of 21 years from the Taibah University, College of Dentistry female section enrolled in preclinical operative dentistry for the academic year 2018/02019 participated in the study. Participation was voluntary and students’ consent and approval to participate in the study were presented by their attendance and participation in all the study sessions. All participating students were novices and inexperienced in operative dentistry. 

### 2.1. Cavity Preparation before HVRS Training

At first, an orientation session was delivered to the students including a short lecture on instrumentation using a high-speed contra-angle hand-piece and fissure bur. A practical demonstration for GV black class I cavity preparation for amalgam restoration on a lower right second molar plastic tooth (DPS–Model teeth 47, Kavo, Warthausen, Germany) was also delivered to the students. 

Class I cavity preparation demonstrations were carried out by the same operative dentistry professor (AF) for all participating students in divided groups of five students each. Immediately after the practical demonstration, all students were asked to perform a class I amalgam cavity preparation in lower right second molar plastic teeth (DPS—Model teeth 47, Kavo, Germany) mounted in basic study models (Kavo, Germany) during the first orientation session. Each student used a high-speed contra-angle hand-piece (Kavo ExperTorque™ E680 L, Warthausen, Germany) and tungsten carbide Fissure bur (Meisinger HM UN 245 009, Germany) for cavity preparation. All the participating students used graduated periodontal probes (MEDESY 548-4 CP 15, Pordenone, Italy) for measuring cavity depth and width and dental explorers (DERBY DD116-23GF, Lucca, Italy) for checking the smoothness and directions of the internal walls of the cavity. The duration of the cavity preparation was recorded for each student. The design features and evaluation criteria for class I cavity preparation for amalgam restorations were explained to the students and shown in Table 1.

### 2.2. Haptic Virtual Reality Simulator (HVRS) Training

**Figure 1:**
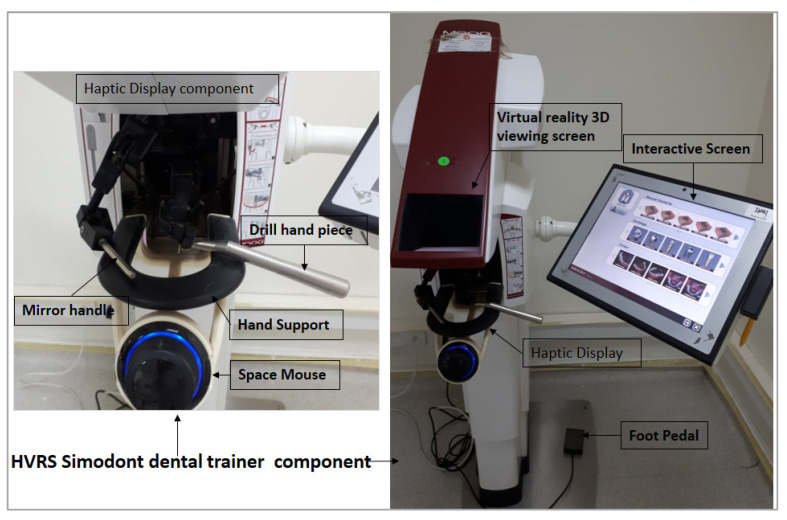
Haptic virtual reality simulator (HVRS) Simodont Dental trainer.

The second orientation session included a lecture and hands-on demonstration delivered to the students on how to use and practice on the HVRS Simodont dental trainer (Moog Industrial Group, Nieuw-Vennep, Netherlands) (Figure 1). The Simodont includes an interactive computer screen and virtual reality 3D viewing screen that shows high-resolution images of teeth, and dental instruments when the student wears stereoscopic glasses. Below the 3D screen, there is a haptic display which is composed of a hand support, mirror handle, and drill hand-piece with virtual tip. The speed of the hand-piece is controlled by a real foot pedal. The hand-piece is connected to a force feedback robotic arm connected to the software giving the haptic tactile sensation feedback of real preparation in enamel and dentine and the voice of the aerator. The Simodont is equipped with courseware software developed by the Academic Centre for Dentistry Amsterdam (ACTA, Amsterdam, Netherlands). The courseware software includes a range of manual dexterity exercises blocks, sound and carious teeth for operative procedures, and different hand and rotary instruments including a choice for right-handed or left-handed users. 

**Figure 2:**
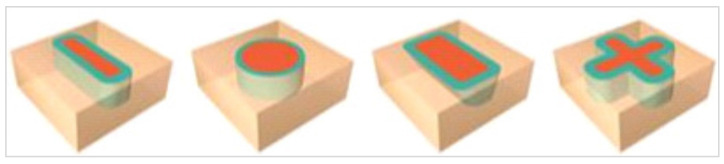
The manual dexterity exercises used in the study.

In this study, the manual dexterity exercises from the courseware package were used to train all students to prepare the basic four shapes illustrated in Figure 2 using a virtual high-speed hand-piece and one type of virtual dental cylinder diamond bur FG 109-010 implemented in the software. The manual dexterity exercise starts with a clear explanation of the assignment with video instructions. The objective of the assignment is the removal of the red area (target) for certain levels; 60%, 75%, 90% without removal of the green area (leeway side and bottom) or beige area (container side and bottom). The software provides the student with objective real time feedback including percentage of removed areas from the target (red), leeway side and bottom (green), and container side and bottom (beige), time elapsed in the exercise, and if student passes or not. Also, the software automatically stores all related data for each students′ trial including; students′ name, academic number, date, start time, elapsed time, drilling time, the removed percentages of the target, leeway side and bottom, container side and bottom, and if the student passes or not. 

The students were instructed to participate on the HVRS Simodont dental trainer on the available manual dexterity exercises in their free time for 20 min/day for four weeks during which no other phantom lab conventional cavity preparation training was delivered or practiced by the students. All students worked independently during HVRS training and faculty members were not present to evaluate or aid the students. The only feedback students received during the HVRS training were from the simulators’ software during their training. After 4 weeks, all related data for each student including the time spent on practice on the simulator were retrieved and collected from the built-in data in the simulator software. 

### 2.3. Cavity Preparation after HVRS Training

The third orientation session was held after 4 weeks from the second orientation session. It included demonstrations for GV black class I cavity preparation for amalgam restoration on the second lower molar plastic tooth. The demonstration was performed by the same operative dentistry professor (AF) for all participating students in divided groups of five students each. Immediately after the practical demonstration, all students were asked to prepare class I amalgam cavity preparation in the lower right second molar plastic tooth with the same instruments used in the first orientation session. The duration of the cavity preparation was recorded for each student.

### 2.4. Assessment and Evaluation

The prepared cavities before and after HVRS training for each student were used as an assessment tool for the students’ psychomotor skills. Two external experienced and calibrated evaluators carried out the assessments. The evaluators were not involved in either the planning of the study or its execution. Each evaluator independently graded each student’s preparation anonymously. The ratings were based on a total score of 16 marks. The average of the ratings of both evaluators determined the final score of the cavity preparation. The cavities were evaluated in accordance with the criteria mentioned in Table 1. The rating marks were; one mark for each correct item, 0.5 for each partially correct item (e.g., mistakes in the cavity preparations which can be corrected such as under-preparation), and 0.0 for each incorrect item (e.g., mistakes in the cavity preparations which cannot be corrected such as over-preparation).

### 2.5. Statistical Analysis

Power calculation was performed for sample size determination. All the data were collected, tabulated, and statistically analyzed using IBM^®^ SPSS^®^ Statistics Version 20 for Windows. Analysis was conducted as means and standard deviations. Intra-class correlation was used to compare between evaluators for inter-evaluator agreement and reliability. Paired *t*-test was used to compare students’ performance before and after HVRS training. A *p*-value of 0.05 was set as a cut-off point to control for alpha error.

## 3. Results

The mean number of HVRS training sessions was 7 with a minimum number of 3 training sessions and a maximum number of 10 sessions. The total mean time of training on HVRS was 208 min with a minimum of 61 min and a maximum of 383 min. The mean time of cavity preparation before HVRS training was 46 minutes with a minimum time of 23 min and a maximum time of 66 minutes. The mean time of cavity preparation after HVRS training was 33 min with a minimum of 12 min and a maximum of 52 min. A statistically significant decrease (*p* < 0.001) in the meantime of the cavity preparation after HVRS training was observed (Table 2). 

**Figure 3:**
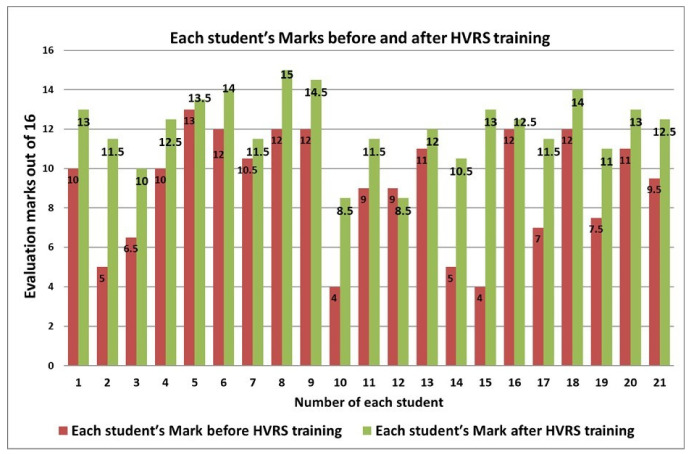
Each Student total mark before and after HVRS training.

**Figure 4:**
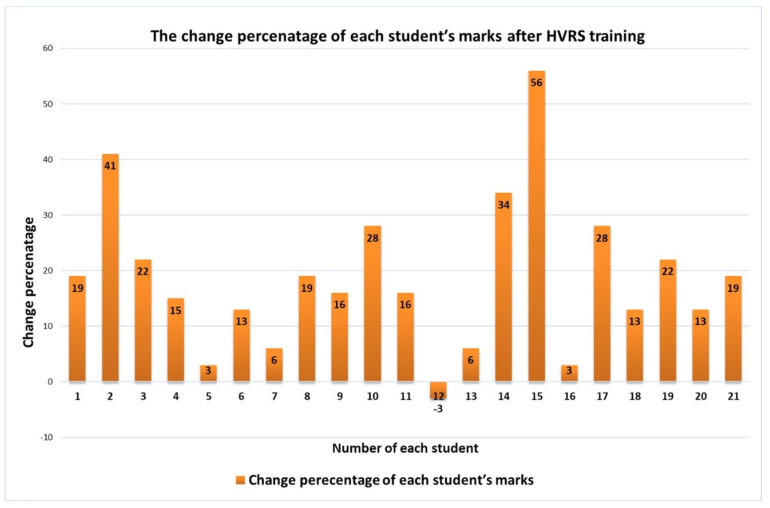
The change of each students′ mark percentage after HVRS training.

Regarding the inter-evaluators agreement of the total evaluation marks of the cavity preparations before and after HVRS training, intra-class correlation showed excellent agreement between the evaluators (ICC 0.978) with a 95% confidence interval of (0.96, 0.988). Regarding the mean of students’ overall marks before and after HVRS training, a statistically significant increase in the mean of total marks after HVRS training was found where there was an increase from 9.1 up to 12.1 (*p* = 0.001) (Table 2). Each students’ overall marks for the cavity preparations before and after HVRS training are displayed in Figure 3. When taking into account the change in students’ performance in the design features of the cavity preparations, there was an improvement in all evaluation criteria scores after HVRS training. The improvements were statistically significant in pulpal floor smoothness (*p* = 0.047), pulpal floor direction (*p* = 0.029), buccal wall direction (*p* = 0.004), lingual wall direction (*p* = 0.025), mesial wall direction (*p* = 0.002), mesial wall smoothness (*p* = 0.01), distal wall smoothness (*p* = 0.001), internal line angle (*p* = 0.024), and internal point angle (*p* = 0.029) (Table 3). The change-percentage improvement of each students’ marks after HVRS training is displayed in Figure 4. 

## 4. Discussion

Manual dexterity is a crucial skill in mastering operative dentistry and a significant portion of undergraduate education is dedicated to training students’ psychomotor clinical skills [8]. Operative dentistry has a long history of using simulation mainly phantom head simulators in preclinical training to learn fine psychomotor skills before the transition to treating real patients. Recently virtual reality simulators with haptic technology have been documented and reviewed as a useful training adjunct tool in operative dentistry [12,13,14,15,16,17,18,19]. It was reported that haptic virtual reality simulators could be an efficient educational tool, as it provides sensory feedback of preparation in enamel and dentine, enabling the students for unlimited practice and repeated attempts to achieve the psychomotor skills without increasing staff demands [12,13,15,19]. Furthermore, it optimizes the acquisition of the basic psychomotor skills required for pre-clinical operative dentistry [12].

In this study, there was a statistically significant increase in the mean of students′ total marks of the cavity preparation together with a statistically significant decrease in the mean of the time consumed in class I amalgam cavity preparation after HVRS training on the Simodont dental trainer. This is consistent with the study by Murbay et al. who found that the students’ group exposed to HVRS training on Simodont performed significantly better in SISTA 1.2 cavity preparation compared to the students’ group not exposed to HVRS training [18]. Furthermore, Al Saud et al. reported an overall improvement of basic manual dexterity skills after HVRS training for all groups at the end of their study demonstrated by lower error scores as well as comparable time for task performance. They further clarify that learning of the basic manual dexterity skills was accelerated when the participants received haptic device feedback in conjunction with experienced dental instructor feedback, compared to the groups which received feedback from the device or instructor only [12]. In this study, a selection of G.V. Black’s Class I cavity preparation to evaluate the students′ psychomotor skill acquisition of cavity preparation after HVRS training was due to its relative complexity in terms of form, depth, wall directions, and smoothness [20]. These types of procedures require mastering the use of instruments via good hand psychomotor skills and eye coordination [17,21]. In our study, not only were the students’ overall marks were considered, but also the change in the students’ performance in specific design features of the cavity preparations were taken into account. This is particularly important to assess if the operative skills acquisition in certain cavity preparation design features would improve using HVRS training or not. Indeed, a significant improvement was found in the pulpal, buccal, lingual, and mesial wall direction, smoothness of the pulpal floor, mesial and distal wall, internal line, and point angles. This is consistent with the study by Murbay et al. who found that students who were trained on HVRS Simodont were able to perform more satisfactory preparations (across several domains including; cavity width and depth, distance from marginal ridge mesially and distally, wall direction, and smoothness, finish of the line and point angles) compared with the group that was not trained on HVRS [18]. Although Vincent et al. 2020 found no significant difference between HVRS trained students group and analogue trained students group in GV black class II cavity preparation, they pointed out that HVRS trained students performed cavity preparation with less iatrogenic damage. In the same context, it was indicated that novice trainees who received a combination of instructor and HVRS Simodont simulator feedback adopted a more cautious strategy, produced fewer errors, and removed less of the target on the manual dexterity exercise on Simodont than those who were exposed to one type of feedback instructor or HVRS feedback alone [22].

In this study, improvement in the novice students′ performance in class I cavity preparation after HVRS training on the Simodont dental trainer may be attributed firstly to the continuous student evaluations provided by the haptic simulators which enhanced the learning of hand-eye coordination and fine psychomotor control [17]. Secondly, the simulator’s visual system enhanced the hand-eye coordination and magnification of the fine details boost the cognitive acquisition of the task and improved the confidence of the novice students [17,23,24]. Thirdly the majority of students preferred to work in 3D vision in the virtual learning environment and performed significantly better in the manual dexterity exercise in a virtual learning environment when working in 3D vision compared with 2D vision [17,25]. Finally, ease of accessibility and availability of the HVRS training to the students at their own pace and after hours without the availability of faculty supervision [11,12,19].

It is worth mentioning that Urbankova in 2010 suggested eight hours of computerized dental simulation training delivered early in the preclinical operative dentistry course to improve students’ performance [26]. Moreover, Vincent et al. pointed out that earlier training on HVRS was effective at improving students’ manual dexterity before experiencing cavity preparation on plastic teeth for the first time [17]. Murbay et al. emphasized the benefits of incorporating HVRS training in preclinical operative dentistry teaching as an adjunct [18]. Al Saud et al. also confirmed that virtual reality haptic simulation helps optimize the acquisition and retention of the basic psychomotor skills required for operative dentistry which is further enhanced when combined with instructor feedback [12]. Nassar and Tekian recommended the integration of conventional training methods with computerized simulation for teaching cavity preparation [19].

It is important to note that this study is single-arm with the intent of assessing the impact of haptic virtual reality simulation on dental students’ psychomotor skills acquisition so that it can be used as an adjunct to phantom head simulators thus reducing training time in the phantom head lab and reducing the number of plastic teeth used. It is not the intention to compare it with phantom head simulators nor to replace it as it is considered the mainstay simulation. Also, this study has a relatively small sample size due to the fact that it was carried out in the female section of the faculty and included all the enrolled female students in preclinical operative dentistry. Furthermore, this sample size was found to be similar to other previous studies [12,18,27]. Nevertheless, the study shows encouraging results indicating that haptic virtual reality dental simulators could be an adjunct for early attainment of the initial psychomotor skills required for operative procedures on artificial acrylic typodont teeth in a conventional phantom head laboratory setting and should be more widely adopted in Saudi Arabia. There is a need for a larger sample size study including both genders and a control group in addition to using digital software evaluation as an adjunct to manual evaluation for the assessment of cavity preparations. 

## 5. Conclusions

Within the limitation of this study, there was an overall improved performance in the psychomotor skills evidenced by improved cavity preparation scores and cavity design features and less time for cavity preparation after HVRS training. It could be beneficial to incorporate HVRS training early in pre-clinical operative dentistry as an adjunct to conventional phantom head training. 

## Figures and Tables

**Table 1:** Design features and evaluation criteria for class I cavity preparation for amalgam restorations.

Class I Cavity Preparation Design Features	Correct 1 Mark	Partially Correct 0.5 Mark	Incorrect 0.0 Mark
**1. Occlusal Outline**	Correct	Partially correct	Incorrect
-Shape: Include all pits, fissures, and angular grooves. Centralized, smooth regular curves.			
-Bucco-lingual width: ¼ inter-cuspal distance 1–1.5 mm, buccal and lingual groove extensions are centralized, extended within 1–1.5 mm from the inter-cuspal distance with 1–1.5 mm mesio-distal width.	Correct	Under extended Less than 1 mm	Over-extended More than 1.5 mm
-Mesio-distal extension: Extends mid-way between apex of fossa and crest of the adjacent mesial/distal marginal ridge.	Correct	Under extended	Over-extended (marginal ridge thickness less than 1.6 mm)
**2. Pulpal floor:**	1.5–2 mm	Less than 1.5 mm	More than 2 mm
-Depth: (1.5–2 mm)			
-Direction: Flat (horizontal) and perpendicular to long axis of the tooth.	Correct	Slightly inclined	Excessively inclined
-Smoothness.	Smooth	Rough	Very rough
**3. Buccal and lingual walls:**	Slightly converge	Straight	Diverge/excessive converge
-Buccal wall direction: Parallel to the corresponding external surface (slightly converge occlusal 2°–5°)			
-Buccal wall smoothness.	Smooth	Rough	Very rough
-Lingual wall direction: Parallel to the corresponding external surface (slightly converge occlusal 2°–5°)	Slightly converge	Straight	Diverge/excessive converge
-Lingual wall smoothness.	Smooth	Rough	Very rough
**4. Buccal and lingual walls:**	Slightly diverge	Straight	Converge/excessive Diverge
-Mesial Wall direction: Parallel to the corresponding external surface (slightly diverge occlusal < 10°)			
-Mesial wall smoothness.	Smooth	Rough	Very rough
-Distal wall direction: Parallel to the corresponding external surface (slightly diverge occlusal <10°)	Slightly diverge	Straight	Converge/excessive diverge
-Distal wall smoothness.	Smooth	Rough	Very rough
**5. Internal line and point angles**	Correct	Sharp	Rough
-Line angle: Definite and smooth			
-Point angle: Definite and smooth	Correct	Sharp	Rough
**Total 16 marks**			

**Table 2:** Mean of the students’ total marks and time of cavity before and after HVRS training.

	Before Training Mean (SD)	After Training Mean (SD)	*p*-Value
Total marks	9.1 (2.9)	12.1 (1.76)	0.001 *
Time of cavity preparation	46.3 (10.5)	33.6 (10.5)	0.001 *

* Statistical significant *p* < 0.05 (SD): Standard Deviation.

**Table 3:** The mean of students’ performance in cavity preparation before and after HVRS training.

Cavity Details	Before Mean (SD)	After Mean (SD)	*p*-Value
Occlusal outline shape	0.74 (0.34)	0.79 (0.34)	0.504
Bucco-lingual extension	0.76 (0.41)	0.79 (0.30)	0.803
Mesio-distal extension	0.57 (0.40)	0.62 (0.31)	0.715
Pulpal floor depth	0.57 (0.29)	0.71 (0.34)	0.162
Pulpal floor direction	0.50 (0.35)	0.69 (0.29)	0.029 *
Pulpal floor smoothness	0.43 (0.36)	0.64 (0.28)	0.047 *
Buccal wall direction	0.64 (0.28)	0.88 (0.22)	0.004 *
Buccal wall smoothness	0.45 (0.35)	0.55 (0.31)	0.296
Lingual wall direction	0.55 (0.31)	0.76 (0.26)	0.025 *
Lingual wall smoothness	0.55 (0.31)	0.64 (0.32)	0.358
Mesial wall direction	0.62 (0.44)	0.95 (0.15)	0.002 *
Mesial wall smoothness	0.43 (0.33)	0.71 (0.30)	0.01 *
Distal wall direction	0.74 (0.41)	0.93 (0.24)	0.057
Distal wall smoothness	0.48 (0.37)	0.76 (0.26)	0.001 *
Internal line angle	0.60 (0.41)	0.86 (0.23)	0.024 *
Internal point angle	0.57 (0.36)	0.81 (0.29)	0.029 *

* Statistical significant *p* < 0.05 (SD): Standard Deviation.

## Data Availability

Available upon request.
